# Mary Seacole and claims of evidence‐based practice and global influence

**DOI:** 10.1002/nop2.32

**Published:** 2015-10-04

**Authors:** Lynn McDonald

**Affiliations:** ^1^ Department of Sociology and AnthropologyUniversity of GuelphGuelphOntarioN1G 2W1Canada

**Keywords:** Crimean War, Florence Nightingale, health care, Mary Seacole, nursing

## Abstract

**Aim:**

The aim of this paper was to explore the contribution of Mary Seacole to nursing and health care, notably in comparison with that of Florence Nightingale.

**Background:**

Much information is available, in print and electronic, that presents Mary Seacole as a nurse, even as a pioneer nurse and leader in public health care. Her own memoir and copious primary sources, show rather than she was a businesswoman, who gave assistance during the Crimean War, mainly to officers. Florence Nightingale's role as the major founder of the nursing profession, a visionary of public health care and key player in advocating ‘environmental’ health, reflected in her own *Notes on Nursing*, is ignored or misconstrued.

**Design:**

Discussion paper.

**Data sources:**

British newspapers of 19th century and *The Times* digital archive; Australian and New Zealand newspaper archives, published memoirs, letters and biographies/autobiographies of Crimean War participants were the major sources.

**Results:**

Careful examination of primary sources, notably digitized newspaper sources, British, Australian and New Zealand, show that the claims for Seacole's ‘global influence’ in nursing do not hold, while her use of ‘practice‐based evidence’ might better be called self‐assessment. Primary sources, moreover, show substantial evidence of Nightingale's contributions to nursing and health care, in Australia, New Zealand, the USA and many countries and the UK much material shows her influence also on hospital safety and health promotion.

## Introduction

The purpose of this paper was to challenge the presentation of Mary Seacole (1805–1881) as a nurse of global influence and significance. That she was a remarkable person who deserves honouring is not in question, but it is questionable that her accomplishments match those of Florence Nightingale (1820–1910). Both of these women became known during the Crimean War (1854–1856), but Seacole's renown was mainly for her business in the Crimea, whose customers were officers. She received ongoing, sympathetic coverage for her bankruptcy and her fine memoir – *Wonderful Adventures of Mrs Seacole in Many Lands*, 1857 – received favourable coverage, in Australia and New Zealand and Britain; however, never as a source on nursing or health care. Nightingale, by contrast, became famous for her work in the Crimean War, her vigorous championing of better conditions for the soldiers, her detailed research on what went wrong at the war hospitals and how to improve them, to her founding of a nursing school and decades of work mentoring nurses, facilitating the introduction of nursing in many countries and making hospitals safer.

Typical presentations today of Nightingale and Seacole go well beyond ordinary ‘revisionism’ in history. Revisionism is appropriate and indeed welcome, when new material comes to light, or new concerns, such as with the extension of human rights to previously excluded or discriminated‐against groups. Yet revisionist authors must also respect sources, giving precedence to reliable primary sources. Secondary sources must be carefully scrutinized, especially in a field when so many have been shown to be faulty; that is, when their claims are examined against primary sources. In the case of Seacole, 10 common myths about her achievements have been challenged with solid primary sources (McDonald 2013), yet the claims continue to be made as if they were credible.

## Background

Mary Seacole is of interest in the UK primarily for her time in the Crimean War. Many myths have been articulated as to how she got there, which usually blame government departments for prejudice, in some cases the head of the nursing team, Florence Nightingale. The authentic story, using Seacole's own memoir and records from the time, is more prosaic. At the start of the Crimean War she was in London attending to her gold mining stocks – having gone there from Panama, where she had a business provisioning men *en route* to the California Gold Rush. After several months of futile efforts, she decided to seek a position as an army nurse, aware that Nightingale and her team had already left (Seacole 1857b p. 78). She sought to join the second team, but it had either left, or was about to leave. Her own memoir specifies that she did not decide to try to go until after learning of the sinking of a major supply ship (Seacole p. 74), news of which was first published in England on 30 November 1854 (The Times 1854). She never submitted the required written application with references, but dropped in informally to various government and war‐related offices, but too late (Seacole pp. 76–79). She arrived in the Crimea in March or April, or possibly later, in 1855.

## Data sources

British: gale.cengage.co.uk/times‐digital‐archive/times‐digital‐archive‐17852006,aspx.

Australian: trove.nla.gov.au/newspaper/result?

New Zealand: Paperspast.natlib.govt.nz/cgi‐bin/paperspast.

## Discussion

### The Crimean War: Seacole and Nightingale compared

When Seacole arrived in the Crimea, she set up a business in a hut, popularly known as ‘Mrs Seacole's’, which was a restaurant/bar/store/takeaway/catering service for officers, with a ‘canteen for the soldiery’ (Seacole p. 114). While getting her business ready to open, she kindly gave out hot tea and lemonade to soldiers waiting on the wharf for transport to the general hospitals, ‘all the doctors would allow me to give’ (Seacole 1857b p. 101). She made her herbal remedies available to all, but the ingredients were unspecified. Her memoir gives three full chapters (13, 14 and 18) to the meals, catering and services she provided to officers, while there is only passing mention of soldiers, but no details of what she provided for them.

In her memoir, Seacole described precisely three occasions when she gave assistance on the battlefield, postbattle: 18 June, 16 August and 8 September 1855 (Seacole pp. 155–61, 164–67 and 169–72). Yet this is frequently made out to be a regular occurrence. A book by nurses, for example, states that she ‘frequently ventured out onto the battlefield, selling goods and caring for wounded soldiers from both sides’ (McAllister and Lowe 2011 p. 26). A book for schoolchildren describes her ‘main job’ as searching ‘the battlefields for wounded and dying men, even while the guns were still firing’ (Castor 1999, p. 34). An encyclopaedia entry has her caring ‘for British soldiers at the battlefront’, and a ‘familiar figure’ transferring ‘casualties from the front’, for which she won ‘decorations’ from three countries (Encyclopedia Britannica 2014).

Both Seacole personally and her business got favourable newspaper coverage in the *The Times* and other newspapers. Many more examples have been added from provincial newspaper archives (Staring‐Derks *et al*. 2015); the first two authors are ‘ambassadors’ for the Seacole statue campaign, the third its vice chair. These authors, however, draw inferences from the coverage that do not survive scrutiny of the sources themselves, nor of other available sources from the time.

Most of the items on Seacole retrieved are mere passing mentions, with no content on nursing, hospitals or health care. That Seacole and her ‘assistant’ were among the ‘first settlers in the purveying line’ was noted in an Australian newspaper story. The commanders‐in‐chief of the British and French armies, Codrington and Pélissier, were said to have been seen in her ‘establishment’, also ‘Billy Russell’, the war correspondent W.H. Russell, ‘and every officer of the three armies’ (Sydney Morning Herald 1856). This is business and socializing, not the running of a hospital for ordinary soldiers.

Seacole was noted as being present for the awarding of the Order of the Bath to senior officers and that she was introduced to the official who made the presentations on behalf of the Queen, Lord Gough (Daily News 1856b). She recorded the event in her own memoir, adding that she sent a cake to army headquarters for it, ‘decorated with banners, flags, etc.,’ (Seacole p. 169). Lord Gough, on the other hand, made a point of calling on Nightingale at her hospital (Rait 1903 vol. 2, p. 335). Some of the newspaper items simply refer to the location of her hut, a known landmark, for example, that a ‘stone struck her door, which is three and a half or four miles from the park’ (The Times 1855c) and that there was a police station nearby (The Times 1855e). Other mentions are of her catering, for example, that she was the only woman at the horse races, where she ‘presided over a sorely invested tentfull of creature comforts’ (The Times 1855d).

The world's first war correspondent, W.H. Russell, was a customer at ‘Mrs Seacole's’, liked her and paid tribute to her kindness to the soldiers. He recounted spotting her on the battlefield, when he was there interviewing soldiers (The Times 1855b). Seacole quoted his compliments in her memoir (Seacole pp. 171–2), for which he also wrote a warm introduction. All this is to Seacole's credit, but repetition of his praise does not make her battlefield visits more frequent or make her into a ‘battlefield nurse’. In the four volumes of Russell's dispatches published later, Seacole appears only once, briefly (Russell 1856 pp. 187–8). When he returned to the Crimea in 1869, escorting the Prince and Princess of Wales on a state visit, their carriages passed near ‘the site of ‘Mother Seacole's’ (Russell 1869 p. 564). A review of a book recounting another return visit noted ‘the heaps of broken bottles by Mrs Seacole's store’ (The Times 1869a).

Russell's mentions of Nightingale, by contrast, are frequent, many of them in considerable detail. His dispatches, as *The Times* coverage generally, relate the terrible conditions she and her nurses faced and her use of *The Times* fund to purchase desperately needed clothing, bedding, supplies, food, etc., for the hospitals. Many stories describe the failings of the War Office which Nightingale had to overcome. *The Times* also printed letters from her and many more about her, from people at the war. A count of mentions in *The Times* shows more than seven for Nightingale for every one of Seacole. There are too many on Nightingale to include in this paper, but they are available on a website: http://www.uoguelph.ca/~cwfn/archival/times-letters-and-mentions.pdf

Seacole's own account of her trip to and time spent in the Crimea are largely of her business, its challenges and the officers it served. Newspaper coverage of her activities similarly was largely of the business and its various problems:

- A short item announced her intended ‘hotel’ for ‘excursion visitors’ (The Times 1855a).
- An American newspaper story described her ‘hotel for travelers’, with the reporter's promise ‘to board with her next year’ (New York Daily Tribune 1855).
- At the first British assault on the Redan, she sold food and drink to spectators, then gave first aid (Seacole p. 157).
- On her second foray onto the battlefield, she gave first aid after selling goods to spectators (Seacole pp. 164–7).
- Seacole is noted, in a memoir, for providing lunch for a cricket match (Astley 1894, p. 145).
- Her third and last foray onto the battlefield – all were postbattle – took place at the second failed British attempt on the Redan (Seacole pp. 169–72).
- For New Year's Day of 1856 she held a party and took plum pudding and mince pies to the nearby Land Transport Corps Hospital (Seacole p. 187).
- In April 1856, the fighting by then long over, Seacole went out on excursions to see the Crimean countryside; a newspaper story recounts Russian soldiers giving her a religious medallion (Aberdeen Journal 1856).
- In May 1856, a newspaper story reports that she made a ‘grand tour’ in the Crimea, that she was able to leave some stock behind her and that she was given ‘a medal’ by the sultan (The Times 1856b); possibly this is a reference to the religious ‘medallion’ above noted.
- In June 1856, when troops were leaving the Crimea, she was cheered by soldiers as they passed by her hut (The Times 1856c).
- A newspaper story in July 1856, as she was leaving the Crimea, said that she planned to establish a store at Aldershot, a British army base (The Times 1856d); she was seen by an Australian fellow passenger on board the *Indus*, which sailed from Constantinople to Marseilles (Argus 1857a).

Coverage of Nightingale's work during the war was extensive and favourable. In *The Times* alone there were over 200 stories or letters on her over the course of the war. The early stories reported her departure, then the terrible conditions the nurses faced at the Barrack Hospital – the lack of bedding, clothing and supplies, overcrowding and poor nutrition, with much on her use of *The Times* Fund and other donations to meet needs.

Coverage in 1855 moved on to Nightingale's trips to the Crimea and its hospitals, her illness, convalescence and return to work. There was much coverage of the improvement in hospital conditions, the work of the Sanitary and Supply Commissions, with Nightingale's assistance. She was much cited in hearings in London of the Roebuck select committee investigating the state of the army, reported in *The Times* and in papers in Melbourne (*Age* 1855), Sydney (Empire 1855, Sydney Morning Herald 1855) and Adelaide (South Australian Register 1855). Stories in 1856 took up improved conditions for soldiers, with the establishment of the ‘Inkermann Cafe’ and reading rooms, then plans for her return to England and her first work back home to address the problems. There are letters by Nightingale to the editor and some to family members they sent on to a paper. She was frequently mentioned in letters to the editor by people who had visited the war hospitals.

### Post‐Crimea, Seacole and Nightingale compared

Back home, Seacole continued to get good coverage, as a minor celebrity, not as a nurse or health care expert. Her bankruptcy hearings were extensively reported, always with great sympathy. That she wore four medals at the first hearing was noted, but not questioned (The Times 1856i). The humour magazine *Punch* published a cartoon in support of fundraising for her (Punch 1856); another cartoon showed her distributing copies of *Punch*, donated by officers, to men in the local hospital. The article accompanying the cartoon related her difficult, but not desperate, financial situation (Punch 1857). A filly named ‘Mrs Seacole’ won a race held at the garrison at Woolwich (The Times 1856g).

Numerous banquets took place on the return of the army. Newspaper sources show that Seacole was present at two of them (The Times 1856e,f). In addition to the well‐known Surrey Gardens festival held in her honour in July 1857, fundraisers were held for her in Australia and New Zealand (Argus 1856).

The situation is different for Nightingale, who attended no Crimean banquets or fundraisers. However, she was toasted and commended at many, for example, in the Edinburgh toast to ‘the British Army, the Navy, our Allies, the Memory of those who fell and Florence Nightingale’ which was followed by a lengthy description of her work by Sir John McNeill (The Times 1856h). She was toasted also in banquets in London, Hereford, Leeds, Portsmouth, Ledbury and Greenock. Further afield she was toasted in Dublin, Paris, Calcutta, Sydney, Melbourne, Adelaide, Auckland and Hobart, Tasmania. In Paris, fundraising was organized for ‘all British residents on the Continent’ (The Times 1856a).

### Visits to naval bases, by Nightingale and Seacole 1857b and 1859

Both Nightingale and Seacole visited naval bases post‐Crimea – Nightingale twice in 1857, Seacole once in 1859 – but with greatly different purposes and results. Nightingale's first trip was to Haslar, which took more than a day; the second was to the large Melville Hospital, with its ‘dispensing rooms, washhouses, receiving rooms’, and Fort Pitt General Hospital. On both trips she was escorted by Sir John Liddell, the director general of the Medical Department of the Navy.

Both visits were reported in detail, the first as follows:

[They] made a minute inspection of the whole establishment, with the organization of which Miss Nightingale was pleased to express her entire approval…. [They] left yesterday morning for Portsmouth, to visit the Military Hospital there. At the latter establishment, Miss Nightingale was received by Dr Bell, medical inspector and principal officer, with the medical staff, who conducted her through the several wards, the cookhouse, Milldam ward and the 20th, 22nd and 97th regimental hospitals, under the same roof, concluding with a visit to the Royal Ordnance Hospital. Both at Haslar and at Portsmouth, Miss Nightingale recognized several of the patients and the medical officers, with whom she chatted freely. After a minute inspection of the Portsmouth Hospital and its dependencies, the ladies returned to London by an early train (The Times 1857a)

Her Chatham visit a few months later was similarly reported, the purpose again noted as the inspection of its ‘several naval and military hospitals’, where there were ‘upwards of 500 patients’:

Miss Nightingale first inspected the Garrison Hospital at Chatham Barracks…. After spending some time in visiting the several wards, in which there are about 300 patients, the party proceeded to Melville Hospital. This large establishment, which adjoins Chatham Dockyard, is used solely for patients belonging to the Navy and the Royal Marines and, in consequence of its proximity to the two large naval establishments at Chatham and Sheerness, wards are generally all occupied. The number of patients now in that hospital is upwards of 200….During her inspection of this hospital, which occupied upwards of two hours. Miss Nightingale frequently expressed her approval of the excellent arrangements adopted for the comfort of the patients. She was much pleased with the size of the wards, which are commodious, light and well‐ventilated; each patient, it was found on enquiry, having 1200 cubic feet of space (The Times 1857b).

Nightingale's views of Melville Hospital were later recounted with pride by the First Lord of the Admiralty, that she had judged it to be ‘one of the most perfect and best arranged establishments’ (The Times 1864c).

Mrs Seacole's visits, on the other hand, were purely social. They show that she was fondly remembered, but give no evidence of nursing or hospital work. A newspaper story reported her visits to ‘several of the military and naval bases doing duty at Sheerness’, with her plans for further visits. Her ‘Crimean celebrity’ was noted, with the ‘hearty and kind welcome from the garrison officers whose quarters she made her home during her stay’. On her visit to Chatham Barracks and Melville Hospital, she was ‘received with the best feelings by the officers and men. Many of the heroes of the Crimea surrounded her and cheered her enthusiastically’ (Daily News 1859).

### The Turkish Medjidie medal: evidence for and against its award to Seacole

The claim has often been made that Seacole won from 1‐4 medals. Many books for schoolchildren portray her wearing them (Huntley 1993, Moorcroft and Magnusson 1998, Malam 1999, Williams 2003, 2009, Ridley 2009). That the Institute of Jamaica owns the Medjidie and the Legion of Honour is sometimes cited as evidence for their having been awarded to her, but the institute acknowledges its total lack of documentation as to their provenance (Greenland 2012, McDonald 2014, p. 158).

More recently, newspaper archives have been said to provide new evidence that Seacole won at least the Turkish Medjidie medal (Staring‐Derks *et al*. 2015, p. 517). The sources they cite, however, lack specifics. The *Daily News* stated that ‘the Turkish government has given Mrs Seacole, of Balaklava, a medal for her services’, and then suggested that ‘no doubt’ a ‘Crimean medal with the Sebastopol clasp would be well bestowed on her’ (Daily News 1856a). No date of award or award ceremony is mentioned in any of these sources. Moreover, since the Medjidie was, in effect, British military medal–nominations were made by senior army officers – Seacole would not have been eligible. Nor was she eligible for the Crimean medal at all, let alone the Sebastopol clasp, for which service throughout the siege was required (she missed the first half of it). Eleven other provincial newspapers are cited in support of the Medjidie claim (Staring‐Derks *et al*. 2015, p. 518), but again not one provided any concrete details.

Given Seacole's published views of Turks, one might wonder why the Turkish government would award her a medal at all. The commander Omar Pacha was a customer for her ‘bottled beer, sherry and champagne’ (Seacole p. 110). Yet she looked down on ordinary Turks, ‘the degenerate descendants of the fierce Arabs, for lacking in ‘pluck’ (p. 106). While they were not as dishonest as the French or Greeks (p. 111), she wrote: they were ‘deliberate, slow and indolent’, given to ‘the sacred duties of eating and praying and getting into out‐of‐the‐way corners at all times of the day to smoke themselves to sleep’ (p. 109).

Seacole wore three medals, but not the Medjidie, for her portrait by Challen and her photograph by Maull. If she had won any medals, it is likely that she would have worn them. However, the evidence is unequivocal that she did not win the Crimean (Mayo 1897). For the French medal, information was received from the Musée de la Légion d'Honneur, that ‘no trace of a nomination’ in her name could be found (Minjollet 2012). A major biographer looked unsuccessfully in War Office records for evidence of medals (Robinson 2005, p. 167). Another author pointed out that the three medals Seacole wore for the Challen portrait were ‘unquestionably hanging from incorrect ribbons’ (Rappaport 2005, p. 62).

Tellingly, Seacole wore no medals in her portrait on the cover of her memoir, nor, in the text, did she claim to have won any. If she had won so much as one, this would have made history – the first woman to be so honoured. Nightingale won no medals – she was not eligible either – but the Queen sent her a brooch in lieu of a medal.

It is known that Seacole wore medals, varying numbers of them, while out walking in London. An assistant surgeon who knew her saw her in Charing Cross wearing the Crimean medal (Reid 1911, p. 13). He, not so incidentally, praised her for her kindness in giving hot tea to soldiers waiting on the Balaklava wharf for transport, but he never called her a nurse or a doctor. He described her store at Kadikoi, near Balaklava, ‘where she sold all sorts of commodities, clothing and articles of food that were luxuries to us’ (Reid 1911, p. 14).

It seems that Seacole did not wear medals at the Surrey Gardens events, or they likely would have been mentioned in the extensive coverage given them. At one, she is noted to have been seated next to Lord Rokeby (Standard 1857), who would have known that they could not have been hers. She was wearing ‘all her decorations’ when she arrived in Antwerp in 1858, but then that article also called her a ‘companion’ of Nightingale and gave her the title of ‘Lady Seacole’ (Morning Chronicle 1858).

The final point on the medals issue is Seacole's failure to mention any in her will. She made detailed dispositions of her goods: the diamond ring of her late husband, her own jewellery, watch, trinkets, ornaments, furniture, pictures, prints, engravings, plate, linen, china and household effects (Leeds Probate Office 1881). Why no medals if she had won any?

### Nursing in the Indian mutiny?

Many authors have told the story of Seacole's interest in going to nurse in the Indian Mutiny. Staring‐Derks *et al*. (2015, pp. 519–20), additionally, claim that recently discovered newspaper stories give greater credence to the possibility. Certainly they uncovered many articles with that suggestion, but the timing does not work. The first violence occurred in March 1857, the first murders of British nationals in May. Yet in a letter to the editor of *Punch* magazine, dated 8 May 1857, Seacole expressed an interest in going to China, or ‘some other shore to which Englishmen go to serve their country’, and the purpose was not specifically nursing, but ‘woman's work’. India is not mentioned (Punch 1857).

A brief note otherwise covering the Surrey Gardens festival describes Seacole telling the Secretary of State for War of her desire to go to India during the Mutiny (*Times* 1857c). Another newspaper reported a former Nightingale associate offering to the East India Company, which then administered India, to take nurses. It is dated 18 July 1857 and the company's answer 22 August 1857, which stated that no women nurses were wanted (Daily News 1857). Thus, one wonders what Seacole had in mind when, in October 1857, she wrote to the military secretary at the War Office, Sir Henry Storks, asking him to forward a letter (contents unknown) to the War Secretary. If it were an offer, it was late. Yet it has been cited as evidence of Seacole's ‘networking’ skills (Staring‐Derks *et al*. 2015, p. 514). It seems rather to demonstrate lack of practical knowledge of conditions in India and the time it would take to get there.

Much is made of the suggestion of the anonymous nurse who had worked with Nightingale that there were 40 women willing and able to go to nurse in India (Staring‐Derks *et al*. 2015, p. 520). It seems implausible, for only two nurses match the description of having nursed for exactly five months: Elizabeth Blake and Margaret Williams (Wellcome Library). Both were judged satisfactory by Nightingale (McDonald 2010, p. 290 and 2014); both were invalided home and neither is known to have nursed subsequently.

### Seacole's *Wonderful Adventures*

Seacole published her famous memoir (Seacole 1857b) largely motivated by the need to make money (the Surrey Gardens fundraisers in July 1856 did not yield much, after expenses were paid). The book was successful and was promptly reprinted when it sold out. It was soon translated into Dutch (Seacole 1857a) and then French (Seacole 1858).

Staring‐Derks *et al*. found 26 reviews (2015, p. 518). One not on their list praised the book, calling its author an ‘itinerant licensed victualler’ and a ‘jolly old soul’, who had not written ‘a line’ of it. Still, she deserved credit for it was *her* life. The ‘little volume’ would give ‘a couple of hours’ amusement’ (Critic 1857):

Some of the provincial newspaper reviews are brief, a bare sentence for one:

We learn from Mrs Seacole's memoirs that the good lady is a native of Jamaica, her father being a Scotchman and her mother a Creole, that she married Mr Seacole, who was of weak health and soon died and afterwards she went to Panama and set up an inn there. Ultimately she found her way to Balaklava (Lancaster Gazette 1857).

Most of the reviews give excerpts, some numerous, evidently chosen for their dramatic appeal. A lengthy review in an Australian newspaper, which also urged readers to contribute to the Seacole fund, was rife with errors of fact. It called war correspondent W.H. Russell ‘Dr Russell’ (Illawarra Mercury 1857), an error that would appear again in obituaries for Seacole, beginning with *The Times* (1881a). A Sydney newspaper gave a one‐paragraph review, with excerpts on ‘picturesque’, ‘cut‐throat, scenes featuring ‘desperadoes’ and lawlessness (Empire 1857). A New Zealand newspaper gave a warm review with excerpts both of Panamian adventures and the Crimea Lyttelton Times 1857).

The book reviews found were *all* favourable. Not one mentioned Seacole's ‘lamentable blunders’, her use of toxic substances or taking of ‘trophies’ or ‘plunder’ from dead soldiers or churches, which are frankly noted in her memoir (Seacole pp. 31, 167 and 174). There is nothing in any of the reviews to demonstrate medical or nursing competence.

#### Racial equality

Seacole is described as an ‘African‐Jamaican’ several times, with an explicit statement that she respected her ‘African‐Jamaican heritage’ (Staring‐Derks *et al*. 2015, pp. 515–16). Yet her own memoir includes not one mention of ‘Africa’ or ‘African’, and the references to her Creole heritage are negative, while those to her Scottish ancestry are positive (Seacole pp. 1–2). She was three quarters white, had a white husband, white business partner and white clientele. She employed black people, including two servants who travelled with her (Seacole pp. 12, 36, 39) and two ‘good‐for‐nothing black cooks’ in the Crimea (Seacole p. 141). Other examples of disparaging remarks are available (McDonald 2014, pp. 192‐5).

Seacole's memoir shows that she believed in racial equality and utterly rejected the white supremacy views of the southern Americans she met in Panama. At the same time, however, she distanced herself from black people. In an admirable putdown of a prejudiced southerner, she said that if her skin ‘had been as dark as any n*****'s(Ed.), I should have been just as happy and as useful’ (Seacole p. 48). Clearly her skin was not as dark. For skin colour for herself she preferred ‘yellow’ (Seacole pp. 27, 34, 78–9), ‘brunette’ or ‘a little brown’ (Seacole p. 4).

### Further newspaper coverage of Seacole

Newspaper coverage of Seacole in the post‐Crimea period continued, but was far less frequent than that for Nightingale and not at all of the same weight or on the same subjects. As before, stories show that she was well remembered, but are not on nursing or health care. One in a Scottish newspaper noted her being injured in an omnibus accident. When she got off she was ‘instantly recognized by an officer and some of the Crimean heroes’, who both offered their assistance and abused the driver for his carelessness (Caldeonian Mercury 1857).

Her return to Jamaica from London in 1859 was noted on a passenger list, where she was listed as a passenger, with others, including ‘seven Sisters of Charity’ (The Times 1859). Staring‐Derks, Staring and Anionwu, however, interpreted this to suggest that she was ‘accompanied by seven Sisters of Charity’ (2015, p. 521), when there was no indication of any connection in the original story.

Another brief note recorded that Seacole, called the ‘famous Crimean camp follower’, was ‘now living at Panama’ (Daily News 1862). An officer en route to Vancouver Island saw her at a hotel there: ‘In this delightful locality we saw the sunny face of Mrs Seacole, of Crimean renown, gadding about with naval officers on leave from the frigate Orlando’ (United Service Magazine 1863). These items show that Seacole was still known enough to be recalled with affection, but nothing more. At the same time, Nightingale was busy analysing health data for India (Vallée 2006) and extending her hospital analysis (Nightingale 1859) for publication as a major book, *Notes on Hospitals* (Nightingale 1863a).

Seacole's return to England in 1865 was noted (The Times 1865). She was seen at a Jamaica‐related trial in London (The Times 1867a). On her death in 1881, there were substantial obituaries, notably and with numerous factual errors, in *The Times* (1881a). There were shorter items on her will and associated legalities. Stories with substance on Seacole are few: the Surrey Gardens Festival received much coverage, but that was focused on the performances and the firm's later financial difficulties (it, too, went bankrupt). Her exchange with Lord Rokeby was published in *The Times*, but it is confined to her financial situation (Rokeby 1856).

When a new fund was established for Seacole in England in 1867, again there were events in its support, both in England and the colonies, for example, a theatrical performance in London's Haymarket (The Times 1867c) and an amateur performance in Wellington, New Zealand (Wanagui Evening Herald 1867). None of this relates to nursing, in contrast with the fundraising done for the Nightingale Fund, which resulted in the establishment of the first nursing school in the world.

### Nightingale's post‐Crimea writing

While Seacole never published more than her memoir, Nightingale began to publish soon after the war and continued to put out full books, articles, letters to the editor and pamphlets for the rest of her life. A list of her publications takes up 12 pages in the major biography of her (Cook 1913 vol. 2 pp. 437–58). In substance they range from groundbreaking research on the high mortality of the Crimean War and how to prevent its recurrence (Nightingale 1858), her famous *Notes on Nursing: What It Is and What It Is Not* (Nightingale 1860a) and *Notes on Hospitals* (1859 and 1863a). Nightingale as well contributed on such challenging issues as midwifery mortality and how to reduce it (Nightingale 1871). Her Collected Works consist of sixteen, substantial, volumes (McDonald 2001). The volume reporting Nightingale's war work is a full 1074 pages (McDonald 2010).

Newspaper coverage of Nightingale post‐Crimea was similarly of serious substantive issues. The *New York Times* did a lengthy overview of her Crimean War research (Clarke 1857). There were articles on her evidence for the Royal Commission on the war (New York Times 1858) and on nursing in India (McDonald 2009, pp. 549–51). Her *Notes on Nursing* (Nightingale 1860a) was serialized in the *Saturday Evening Post* from March to May in 1860 and published in an American edition (Nightingale 1860d). It appeared in numerous translations: Italian (Nightingale 1860b); German (Nightingale 1860c), Swedish (Nightingale 1861), French (Nightingale 1863b) and Dutch (Nightingale 1863c). She published a short work on deaconess work in Syria (Nightingale 1862).

Nightingale's *Notes on Hospitals* was used by both sides in the American Civil War (McDonald 2011, pp. 592–603). The American Sanitary Commission recognized her as a source for its work (The Times 1867b). Her Crimean experience was said to have inspired American women to nurse in that war (The Times 1864b).

By 1863 Nightingale was researching on India for another Royal Commission (Vallée 2006, pp. 130–83). This work would go on for many years and became a major lifetime contribution. That same year, 1863, she established a relief fund for Polish patriots (The Times 1863a), while a paper she did on statistics of surgical outcomes was given at a conference in Berlin (Nightingale 1863a appendix). The surgical outcomes paper was part of her endeavour to standardize hospital statistics to enable comparative analysis of outcomes.

Her paper on native colonial schools and hospitals (Nightingale 1863d) was read at the Social Science Congress in Edinburgh, where it got newspaper coverage, notably because the Queen's son, Prince Alfred, attended the session (The Times 1864d). The event was picked up in due course in Australian newspapers (Courier 1863, South Australia Weekly Chronicle 1863, Star 1863). Nightingale made a significant start on this enormously difficult issue. The newspaper coverage shows that her ideas were taken seriously, but there would be no concrete results. She sent a further paper on the aboriginal issue to the next meetings (The Times 1864d). Nightingale herself gave up on the Colonial Office, to concentrate her efforts on India, where she found she could get results, albeit always too slow and never vigorous enough.

Nightingale advised the British delegation sent to the founding meeting for what became the Geneva Convention on the treatment of prisoners of war (News Review of British Red Cross Society 1859). The original convention, however, was confined to voluntary assistance in war, on which her position was sceptical. Nightingale feared that the use of volunteers for such essential tasks as removing the wounded from the battlefield and providing early treatment made war ‘cheap’. Armies should be made to pay the full costs of any war they undertook.

In 1864, Nightingale met with the Italian independence leader, General Garibaldi (The Times 1864a). Again, the story was picked up by Australian newspapers (Sydney Morning Herald 1864, Sydney Mail 1864). This meeting shows her deep fondness for the country of her birth. As to Garibaldi himself, however, great man that he was, he had no ideas of public health or public administration. Nightingale's work to institute trained nursing in Sweden began in 1865 (McDonald 2009, pp. 444–50). Sweden was one of the first European countries to take up her principles of nursing, but she herself had little subsequent contact with Swedish nursing leaders.

The next year, on the outbreak of the Austro‐Prussian War, she assisted on relief efforts (McDonald 2011, pp. 612–27). This war can be seen, with the benefit of hindsight, as a precursor to the much larger Franco‐Prussian War, on which she did considerable work. Nightingale published on Italian independence also that year (Illustrated London News 1866).

Her considerable efforts to establish trained nursing in Australia began in 1867 (McDonald 2009, pp. 409–32). The nurses actually started nursing in Sydney in 1868, when one of their first patients was Prince Alfred, who had been wounded in an assassination attempt (The Times 1868). There was substantial newspaper coverage in Australia and also in New Zealand, for years on the various stages from planning to sending the nurses and their spread throughout the colonies.

By 1869, stories began to appear on the use of Nightingale's advice on barracks in India (The Times 1869b). The barrack improvement work derives from the Royal Commission on India, subsequently followed up by the Army Sanitary Commission. The following year Nightingale published on Indian sanitation in a Bengal journal (Nightingale 1870). This was her first venture in publishing in a journal run by Indian nationals. She would increasingly work with Indian nationals on public health and related social reform issues.

During the Franco‐Prussian War of 1870–71, Nightingale worked strenuously on relief work (McDonald 2011, pp. 627–822). As conditions became desperate in France, which lost the war, she appealed for support for the starving civilian population (The Times 1871). Her appeal was reprinted in the U.S. (New York Times 1871). Nightingale's sympathies were clearly with France in this terrible war, although France had been the instigator. A great complication, the Crown Princess of Prussia was the Princess Royal, daughter of Queen Victoria and an able woman committed to improving nursing in her adopted country. Only one Nightingale nurse nursed in that war, Florence Lees, who happened to be in France at the time and who was fluent in both French and German. The National Aid Committee did not send nurses, but Nightingale acted as intermediary with the Crown Princess.

In 1872, the founder of the International Red Cross, Henri Dunant, credited Nightingale with the inspiration for the Geneva Convention (The Times 1872a). She was decorated by both France and Prussia for her assistance during the Franco‐Prussian war (McDonald 2011, p. 822). Most of her work on the war was in support of the National Aid Society, the original Red Cross in the U.K. When it decided not to send nurses, Nightingale's efforts were confined to the organization of medical assistance and, for the French, relief as famine resulted from the Prussian siege. Nightingale also gave what assistance she could to people acting independently of the aid society.

The year 1872 marks also Nightingale's first work on establishing trained nursing in the United States, beginning with Bellevue Hospital, New York (McDonald 2009, pp. 499–508). American nursing followed her model closely, but with little direct involvement of Nightingale herself. She corresponded to Harriet Beecher Stowe, the famous author of *Uncle Tom's Cabin,* on nurse training for the U.S. (McDonald 2009, pp. 800–8). However, Stowe did not pursue the subject. Still in 1872, she wrote in support of education for women in Germany (The Times 1872b) and advised on nursing in Hesse‐Darmstadt (McDonald 2009, pp. 453–60). The Grand Duchess of Hesse‐Darmstadt was Princess Alice, another daughter of Queen Victoria keen to advance trained nursing.

In 1873, Nightingale's paper on water and health in India was read at a meeting of the National Association for the Promotion of Social Science (The Times 1873). Water and health issues would continue to be major concerns in her India work. She published also that year on prisons in an American newspaper (Nightingale 1873), an early interest, but not one she actively pursued.

In 1874, Nightingale published on irrigation in India (Illustrated London News 1874 and Nightingale 1874), following up her 1873 work on water. In 1874, also, she began work on nurse training at Boston (McDonald 2009, pp. 498–510). She sent the first trained nurses to Montreal in 1875 (McDonald 2009, pp. 528–46). The Montreal work, but not Boston, would become a major endeavour, entailing the selection and briefing of the matron and nurses, ongoing contact with them as problems developed and liaison with hospital administrators and an architect on plans for a new hospital in Montreal, which in fact was never built.

In 1876–77, Nightingale supported refugee relief for Bosnia and Bulgaria (The Times 1876a, 1876b). Her letter to Gladstone on Bosnia was read out at a Birmingham Liberal meeting (The Times 1877a); This refugee work was done in conjunction with a family friend who was deeply committed to the work and spent time in Bosnia, but who needed considerable help with newspaper publicity, fundraising and brochures.

Indian irrigation and famine relief were major topics in 1877 (Nightingale 1877 and The Times 1877b). That year also she mentored the first American trained nurse, Linda Richards (McDonald 2009, pp. 511–2). Richards would be a major force in spreading the Nightingale method throughout the United States and later also to Japan. Nightingale also in 1877 wrote a tribute on the death of Dr Samuel Gridley Howe, a leading American doctor who had early on encouraged her vocation (Richards 1934, pp. 146–7). She supported missionary nursing in Madagascar with a fundraising letter in *The Times* (McDonald 2009, pp. 553–4). Nightingale's contact with the bishop who organized the fund was through a nurse, Emily Gregory, with whom Nightingale kept in touch after she left the school.

Still in 1877, Nightingale sent a message of support to an international conference on ending government regulation of prostitution (Butler 1877). Years earlier, she had managed to delay, but not stop, British legislation on the subject, the Contagious Diseases Acts of 1864 and 1866, and would continue to work for repeal. This international initiative was headed by Josephine Butler, while Nightingale played a supportive role.

In 1878, Nightingale again published on famine in India in two British periodicals (Nightingale 1878a, 1878b). The fact of periodic famines led her to work on prevention and relief, which meant attention to water supply and irrigation, for drought was usually the cause of famine. In 1879, she published on the work of a missionary health officer in India (Nightingale 1879). Famine was again the core concern. She worked on nursing and relief assistance for the Zulu War (McDonald 2011, pp. 852–7).

In 1880, Nightingale published on ‘woman slavery’ for the Aborigines’ Protection Society (Nightingale 1880). This was prompted by reports of women being sold for cattle in Natal, South Africa. Such sales were allowed by British courts as part of ‘native law’. She next assisted on providing nursing for the Transvaal War, also known as the First Boer War (McDonald 2011, pp. 857–74). There were by then trained and experienced nurses to send, but the supposedly trained male orderlies turned out to be both incompetent and frequently drunk. For Nightingale, it was the mistakes of the Crimean War, ‘over again’.

In 1881, Nightingale gave her attention to Indian tenancy reform (Sen 1937). This shows her broadening her analysis: ill health among the landless peasantry was often the result of extreme poverty, hence the interest in credit and land ownership. American doctors visiting London in 1881 praised the new Marylebone Workhouse Infirmary, a hospital for which Nightingale advised on the design (The Times 1881b). Nightingale's advice on hospital design typically devolved on efficiency in the wards, saving nurses unnecessary effort, while for the nurses’ home privacy and comfort were the main subjects.

The above examples give only brief indications of the international impact of Nightingale's work, especially in nursing. Further examples are available in a Timeline (McDonald 2013). Altogether it is clear that, for the same years as international information was obtained on Seacole, the breadth and significance of Nightingale's international work is remarkable.

### Australian and New Zealand coverage of Nightingale's work and influence

While Australian and New Zealand newspapers reported the fundraising activities for Seacole (Staring‐Derks et al. 2015, pp. 519–20), fundraising activities for Nightingale were far greater and newspaper coverage reflects this. A story in *The Times* noted that a ‘considerable’ number of meetings had been held ‘in all the colonies in support of the fund’ (The Times 1856j). In South Australia, every post office took contributions (South Australian Register 1857), in Victoria every bank (Lyttelton Times 1856). There were numerous reports on the fund in Tasmania (Courier 1856).

Many people in Melbourne wanted the money raised to be spent locally, but it was agreed that there was ‘no institution’ there where a training school could be started, so that the money went to the general fund in London (Argus 1857b). Coverage of these meetings included serious discussion of Nightingale's work during the war and the desirability of her continuing it by founding a nursing school.

Nightingale was to be reminded of the Australians’ generosity in 1863, when Lady Dowling, wife of the chief justice of New South Wales, wrote to her about the need to get trained nurses into Australian hospitals. Nightingale then set about to get a team of matron and nurses sent out, who began work in 1868 (McDonald 2009, pp. 405–17).

New Zealanders contributed to the Seacole Fund and to the Nightingale Fund, but the earliest trained nurses to work in New Zealand had a Nightingale connection: one, E. Ward, had trained in midwifery at the ‘Florence Nightingale Ward’ at King's College Hospital (Clarke 2012 p. 41) and Mary Tattersall, a nurse who was well regarded by Nightingale (Mary Tattersall 2014 and McDonald 2010, p. 465).

There are stories in over 75 Australian, New Zealand and Tasmanian newspapers 1855–81 on Nightingale's work. A major newspaper, the *Sydney Morning Herald*, published some 150 stories over that period, on her sending out nurses, with their subsequent appointments as they moved on to other hospitals. The construction of the new Sydney Infirmary and nurses’ home was amply covered. Reviews of and excerpts from, her writing, appeared from *Notes on Nursing*,* Notes on Hospitals*, ‘Una and the Lion’ and her substantial Indian report. Her two papers on particular Australian issues were given much coverage: on native colonial schools and hospitals and the disappearance of aboriginal races.

Not only was her work during the Crimean War extensively reported, so were her efforts on later wars, notably the American Civil War and the Franco‐Prussian War. There is simply nothing equivalent on Seacole, for whom coverage showed affection and respect, but no contribution to nursing, hospitals or health care.

### Seacole: blunders, embellishments and omissions

Proponents of Seacole as a leading nurse simply ignore evidence to the contrary, both that in her own book and in other sources of the time. She admitted using toxic substances in her cholera ‘remedy’, but thought that they were effective. Indeed, the only remedy for which she gave specific ingredients was for cholera, to which she added, for ‘stubborn cases’, ‘ten grains of sugar of lead, mixed in a pint of water, in doses of a tablespoonful every quarter of an hour’ (Seacole p. 31).

In a puzzling admission, Seacole held that ‘few constitutions permitted the use of exactly similar remedies and that the course of treatment which saved one man would, if persisted in, have very likely killed his brother’ (Seacole p. 32). However, for cholera and other bowel diseases, the same treatment does work: oral rehydration therapy, discovered only in the 1960s. Seacole's ‘remedy’ not only contained substances that are toxic in any dose, lead acetate and mercury chloride, but they and such other ingredients as mustard poultices and emetics, dehydrate the patient. And, while Seacole claimed some successes, she also acknowledged ‘lamentable blunders’ in her remedies, even that, when she later came across notes of her treatments, she was made to ‘shudder’ (Seacole p. 31). Yet her supporters continue to claim that she learnt ‘practice‐based evidence’ and acquired ‘Western medical knowledge’ from European doctors in Jamaica (Staring‐Derks *et al*. 2015, 516). No independent assessment of either her diagnostic skills or remedies exists. The ‘evidence’ pertains only to her own opinions of her practice.

Doctors’ memoirs and letters from the period show only that they credited her with kindness, not nursing or medical skills. One who served with the Turkish Army referred to her having a ‘store…where, in an emergency, one can obtain a meal’ (Buzzard 1915, p. 179). Another, noting her ‘store at Kadikoi’, called her ‘one of the many sutlers or camp followers who sold goods (mostly food and drink) to the troops’ (Bonham‐Carter 1868, p. 157). By the time of the English 1866 cholera epidemic, Seacole was back in London. A newspaper story lists her contribution to the Mansion House Cholera Relief Fund as ‘100 bottles of anti‐cholera medicine and 100 boxes of pills’ (The Times 1866). No ingredients or doses are specified.

It would be unfair to fault Seacole for her mistaken cholera remedy when so many doctors used the same or similar ingredients (McDonald 2013, 2014, pp. 47–9). However, it is noteworthy that British Army doctors during the Crimean War became critical of them. Reports they sent back to the Army Medical Department state unambiguously that they tried ‘varied’ treatments ‘without success’, and that ‘in no instance’ of recovery did ‘any plan of treatment’ prove to be any more advantageous than any other (Smith 1858 vol. 2, p. 261). Seacole was, with many people, wrong on cholera and bowel diseases, hardly an ‘exemplar of a global nursing role model’ (Staring‐Derks *et al*. 2015, p. 515).

Authors on Seacole typically omit mention of her admitted ‘blunders’, or that she took a ‘horsewhip’ to her young, neglectful, mule attendant (Seacole p. 158), cut souvenir buttons off the coats of dead Russians and accepted ‘plunder’ and ‘trophies’ stolen by soldiers from Russian churches (Seacole pp. 167 and 174).

## Conclusion

### The real ‘global nurse’

The evidence shows that the serious work to found the profession of nursing was led by Florence Nightingale, who did major work also on hospital safety and the institution of public health care. Post‐Crimea, she conducted research to determine how to reduce death rates, then founded the first nurse training school in the world, which in time sent out trained nurses and matrons to establish professional nursing in many countries. Without ever going to India, she worked for decades on improving health care there, along with social and political reform, famine prevention and relief. She documented the high rates of morbidity and death in colonial aboriginal schools and hospitals. Her work was global and it was globally influential.

Primary sources on Seacole have shown wide interest in her life and circumstances, but not any contributions to nursing or health care. International coverage is similar to that in the U.K. Her book was favourably discussed, wherever it was reviewed, but nowhere treated as more than enjoyable reading. Excerpts appear to have been selected for general interest. By contrast, international coverage of Nightingale over the same period shows her to have been taken as a major contributor to the establishment of nursing and the reform of hospitals and a vigorous advocate of broader social and health care reforms, both for the military and the civil population. There was much coverage also of her work on such other concerns as the vote for women, health in India and relief in numerous famines and wars. Seacole should be credited for the contribution she actually made and Nightingale for hers.

Finally, there are lessons to be drawn from the analysis about the reinterpretation of people's contributions over time, or revisionism. The tendency to consider that all people in a particular era share the same values and prejudices is overly simplistic. Nightingale was white and of a privileged family background, but she was highly critical of British imperialism and views of racial supremacy. Her papers on aboriginal peoples show this. Her work on Indian health concerns was accompanied by support for Indian nationals; for example, she wrote a campaign letter for the first Indian to be elected to Parliament.

Not everyone shares the prevailing set of attitudes and some indeed oppose them. Clearly there would be no social change if this were not so. Change requires leaders both of the privileged group and the one seeking recognition‐independence‐equal rights. Nightingale, thanks to her family, was especially conscious of race issues – her grandfather was a leading abolitionist. She can be seen as carrying those concerns forward, indeed as an early contributor to anti‐racism.

## Conflict of interest

No evident conflict of interest.

## Author contributions

The (sole) author conducted all the research and wrote the paper.

- substantial contributions to conception and design, acquisition of data, or analysis and interpretation of data;
- drafting the article or revising it critically for important intellectual content.

## References

[nop232-bib-0001] *Aberdeen Journal* (1856) The East, 2 April, p. 3F.

[nop232-bib-0002] *Argus* (1856) The Nightingale Fund, 22 August, p. 2.

[nop232-bib-0003] *Argus* (1857a) Mother Seacole, 3 September, p. 7.

[nop232-bib-0004] *Argus* (1857b) Untitled, 9 November, p. 5.

[nop232-bib-0005] Astley J.D. (1894) Fifty Years of my Life in the World of Sport, Vol. 2. Hurst & Blackett, London.

[nop232-bib-0006] Bonham‐Carter V. , ed. (1868) Surgeon in the Crimea: The Experiences of George Lawson Recorded in Letters to his Family 1854‐55. Constable, London.

[nop232-bib-0007] Butler J. (1877) Letter to Nightingale 5 June, Woodward Biomedical Library B.24.

[nop232-bib-0008] Buzzard T. (1915) With the Turkish Army in the Crimea and Asia Minor: A Personal Narrative. John Murray, London.

[nop232-bib-0009] *Caledonian Mercury* (1857) Home Intelligence, 1 August.

[nop232-bib-0010] Castor H. (1999) Mary Seacole Franklin Watts, New York.

[nop232-bib-0011] Clarke A. (2012) Born to a Changing World: Childbirth in Nineteenth‐Century New Zealand Bridget Williams Books, Wellington.

[nop232-bib-0012] Clarke M.C. (1857) Florence Nightingale. New York Times, 18 December, p. 1A‐F.

[nop232-bib-0013] Cook E.T. (1913) The Life of Florence Nightingale, Vol. 2. Macmillan, London.

[nop232-bib-0014] *Courier* (1856) The Nightingale Fund, 16 July, p. 2.

[nop232-bib-0015] *Courier* (1863) The Social Science Congress at Edinburgh, 25 December, p. 3.

[nop232-bib-0016] *Critic* (1857) Untitled. 16,391 15 July, pp. 321–2.

[nop232-bib-0017] *Daily News* (1856a) Our Army in the East, 2 May.

[nop232-bib-0018] *Daily News* (1856b) Movements of the Army in the Crimea, 7 June.

[nop232-bib-0019] *Daily News* (1857) Mrs Seacole, 31 July.

[nop232-bib-0020] *Daily News* (1859) Naval and Military, 2 February 1859.

[nop232-bib-0021] *Daily News* (1862) Untitled, 17 April.

[nop232-bib-0023] *Empire* (1855) Mr Sidney Herbert Before Mr Roebuck's Committee, 30 July, p. 2.

[nop232-bib-0025] *Empire* (1857) The Wonderful Adventures of Mother Seacole, 4 November, p. 3.

[nop232-bib-0026] *Encyclopedia Britannica* (2014) Mary Seacole. 973210/Mary‐Seacole. Retrieved from http://www.britannica.com/EBchecked/topic on 01 December 2014.

[nop232-bib-0027] Greenland J. (2012) Email to author, 10 December.

[nop232-bib-0028] Huntley E.L. (1993) Two Lives: Florence Nightingale and Mary Seacole Bogle‐L'Ouverture, London.

[nop232-bib-0029] *Illawarra Mercury* (1857) The Mother Seacole Testimonial, 16 November, p. 1.

[nop232-bib-0030] *Illustrated London News* (1866) Garibaldians in Florence, 5737, 16 June, p. 37.

[nop232-bib-0031] *Illustrated London News* (1874) Irrigation and Means of Transit in India, 30 July, p. 99.

[nop232-bib-0032] *Lancaster Gazette and General Advertiser for Lancashire, Westmorland, Yorkshire, &c* (1857) Untitled, 1 August, p. 6.

[nop232-bib-0033] Leeds Probate Office (1881) Last will and testament of Mary Seacole, 11 July.

[nop232-bib-0034] *Lyttelton Times* (1856) Testimonial to Miss Nightingale, 20 August, p. 4.

[nop232-bib-0035] *Lyttelton Times* (1857) Extracts, 2 December, p. 3.

[nop232-bib-0036] Malam J. (1999) Mary Seacole. Evans Brothers, London.

[nop232-bib-0037] Mary Tattersall (2014) New Zealand Crimean War Veterans. Retrieved from http://crimeanwarveterans.blogspot.ca/2014/01/tattersall-mary.html on 01 December 2014.

[nop232-bib-0038] Mayo J.H. (1897) Medals and Decorations of the British Army and Navy. Archibald Constable Vol. 2 Crimea 1854‐6, London.

[nop232-bib-0039] McAllister M. & Lowe J.B. (2011) The Resilient Nurse: Empowering Your Practice Springer, New York.

[nop232-bib-0040] McDonald L. , ed. (2001 –12) The Collected Works of Florence Nightingale, Vol. 16. Wilfrid Laurier University Press, Waterloo.

[nop232-bib-0041] McDonald L., ed. (2009) Florence Nightingale on Extending Nursing Wilfrid Laurier University Press, Waterloo.

[nop232-bib-0042] McDonald L., ed. (2010) Florence Nightingale and the Crimean War Wilfrid Laurier University Press, Waterloo.

[nop232-bib-0043] McDonald L., ed. (2011) Florence Nightingale on Wars and the War Office Wilfrid Laurier University Press, Waterloo.

[nop232-bib-0044] McDonald L. (2013) Florence Nightingale and Mary Seacole on Nursing and Health Care. J Adv Nurs. 69(11), 1436–44. 24224570 10.1111/jan.12291

[nop232-bib-0045] McDonald L. (2014) Mary Seacole: The Making of the Myth Iguana Books, Toronto.

[nop232-bib-0046] Minjollet C. (2012) Email from Musée de la Légion d'Honneur to G. Vallée, 4 September.

[nop232-bib-0047] Moorcroft C. & Magnusson M. (1998) Mary Seacole 1805‐1881. Channel Four Learning, London.

[nop232-bib-0048] *Morning Chronicle* (1858) Promotion of Mrs Seacole, 14 June.

[nop232-bib-0049] *New York Daily Tribune* (1855) An Excursion to Sevastopol, 18 June. p. 3.

[nop232-bib-0050] *New York Times* (1858) Florence Nightingale Before the Army Medical Reform Commission, 11 March.

[nop232-bib-0051] *New York Times* (1871) Letter from Florence Nightingale, 28 February, p. 6.

[nop232-bib-0052] *News Review of British Red Cross Society* (1859) Untitled, April 1959, p. 58.

[nop232-bib-0053] Nightingale F. (1858) Notes on Matters Affecting the Health, Efficiency and Hospital Administration of the British Army Harrison, London.

[nop232-bib-0054] Nightingale F. (1859) Notes on Hospitals, Being Two Papers read before the National Association for the Promotion of Social Science John Parker, London.

[nop232-bib-0055] Nightingale F. (1860a) Notes on Nursing: What It Is and What It Is Not Harrison, London.

[nop232-bib-0056] Nightingale F. (1860b) Cenni sull'assistenza degli ammalati, Quello che i assistenza, e quello che non, trans Sabilla Novello. Fratelli Bocca, Turin.

[nop232-bib-0057] Nightingale F. (1860c) Anmerkungen zür Krankenpflege: Was Krankenpflege ist, und was sie nicht ist, trans Adolph Wiesner and Friedrich Eiche. Nicolaus Muller, New York.

[nop232-bib-0058] Nightingale F. (1860d) Notes on Nursing: What It Is and What It Is Not D. Appleton, New York.

[nop232-bib-0059] Nightingale F. (1861) Om sjuksketsel: hvad den icke den är, och hvad den icke är. Götebrg: Lamm Göteborg.

[nop232-bib-0060] Nightingale F. (1862) Deaconesses Work in Syria: Appeal on Behalf of the Kaiserswerth Deaconesses’ Orphanage at Beyrout. London.

[nop232-bib-0061] Nightingale F. (1863a) Notes on Hospitals, 3rd edn Longman, Green, London.

[nop232-bib-0062] Nightingale F. (1863b) Des soins à donner aux malades: ce qu'il faut faire, ce qu'il faut éviter. Didier, Paris.

[nop232-bib-0063] Nightingale F. (1863c) Over Ziekenverpleging, trans. Mevr Busken Huet. J.J. Weeveringh, Haarlem.

[nop232-bib-0064] Nightingale F. (1863d) Colonial Schools and Hospitals. Transactions of the National Association for the Promotion of Social Science. London, pp. 475–88 and 501–10.

[nop232-bib-0065] Nightingale F. (1870) Transactions of the Bengal Social Science Association 4. pp. xiv–xv and 1–9.

[nop232-bib-0066] Nightingale F. (1871) Introductory Notes on Lying‐in Institutions Longmans, Green, London.

[nop232-bib-0067] Nightingale F. (1873) Florence Nightingale on Prison Discipline. Hartford Courant 11 October, p. 11.

[nop232-bib-0068] Nightingale F. (1874) Life or Death by Irrigation. Harrison & Sons, London.

[nop232-bib-0069] Nightingale F. (1877) Irrigation and Water Transit in Madras, 29 June. Illustrated London News.

[nop232-bib-0070] Nightingale F. (1878a) A Water Arrival in India, Good Words, July, pp. 493–6.

[nop232-bib-0071] Nightingale F. (1878b) The People of India. Nineteenth Century 4(18) August, 193–221.

[nop232-bib-0072] Nightingale F. (1879) A Missionary Health Officer in India. Good Words pp. 492–6, 565‐71, 635‐40.

[nop232-bib-0073] Nightingale F. (1880) Woman Slavery in Natal. Transactions of the Aborigines’ Protection Society. Aborigines’ Protection Society, London, pp. 222–223.

[nop232-bib-0074] *Punch* (1856) A Stir for Seacole, 6 December, p. 221.

[nop232-bib-0075] *Punch* (1857) Our own Vivandière, 30 May, p. 102.

[nop232-bib-0076] Rait R.S. (1903) The Life and Campaigns of Hugh, First Viscount Gough, Field Marshal, Vols. 2. Archibald Constable, London.

[nop232-bib-0077] Rappaport H. (2005) Helen Rappaport Replies. History Today 55(3), 62.

[nop232-bib-0078] Reid D.A. (1911) Memories of the Crimean War: January 1855 to June 1856 St Catherine Press, London.

[nop232-bib-0079] Richards L.E. (1934) Samuel Gridley Howe Appleton, New York, pp. 146–7.

[nop232-bib-0080] Ridley S. (2009) Mary Seacole and the Crimean War Franklin Watts, London.

[nop232-bib-0081] Robinson J. (2005) Mary Seacole: The Charismatic Black Nurse Who Became a Heroine of the Crimea Constable, London.

[nop232-bib-0082] Rokeby (1856) Letter to the editor. Times, 25 November, p. 6F.

[nop232-bib-0083] Russell W.H. (1856) The War: From the Death of Lord Raglan to the Evacuation of the Crimea G. Routledge, London.

[nop232-bib-0084] Russell W.H. (1869) A Diary in the East, During the Tour of the Prince and Princess of Wales G. Routledge, London.

[nop232-bib-0085] Seacole M. (1857a) Wonderful Adventures of Mrs Seacole in Many Lands, ed. W.J.S. James Blackwood, London.

[nop232-bib-0086] Seacole M. (1857b) Mary Seacole's Avonturen in de West en in de Krim. P.C. Hoog: Rotterdam.

[nop232-bib-0087] Seacole M. (1858) Aventures et voyages d'une Créole à Panama et en Crimée, trans. Victorine Rilliet de Constant Massé. Lausanne: Delafontaine.

[nop232-bib-0088] Sen P., ed. (1937) Florence Nightingale's Indian Letters: A Glimpse into the Agitation for Tenancy Reform, Bengal 1878–82 Mihir Kumar Sen, Calcutta.

[nop232-bib-0089] Smith A. (1858) Medical and Surgical History of the British Army which Served in Turkey and the Crimea, Vols 2. Harrison, London.

[nop232-bib-0090] *South Australia Weekly Chronicle* (1863) Our London news letter, 19 December, p. 4.

[nop232-bib-0091] *South Australian Register* (1855) Mr Roebuck's Committee, 16 June, p. 3.

[nop232-bib-0092] South Australian Register (1857) The Nightingale Testimonials, 22 August, p. 2.

[nop232-bib-0093] Standard (1857) Benefit of Mrs Seacole, 28 July, p. 7.

[nop232-bib-0094] *Star* (1863) Social Science Congress at Edinburgh, 17 December, p. 2.

[nop232-bib-0095] Staring‐Derks C. , Staring J. & Anionwu E.N. (2015) Mary Seacole: global nurse extraordinaire. Journal of Advanced Nursing 71(3), 514–524. 25382171 10.1111/jan.12559

[nop232-bib-0096] *Sydney Mail* (1864) Arrival of Garibaldi in England, 18 June, p. 3.

[nop232-bib-0097] *Sydney Morning Herald* (1855) Mr Roebuck's Committee, 6 July, p. 3.

[nop232-bib-0098] *Sydney Morning Herald* (1856) From our London Correspondent, 8 December.

[nop232-bib-0099] *Sydney Morning Herald* (1864) Week ended April 16, 16 June, p. 2.

[nop232-bib-0100] *Times* (1854) The Gale in the Black Sea, 30 November, p. 7A.

[nop232-bib-0101] *Times* (1855a) The Siege of Sebastopol, 9 March, p. 9E.

[nop232-bib-0102] *Times* (1855b) The Fall of Sebastopol, 26 September, p. 6F.

[nop232-bib-0103] *Times* (1855c) The British Expedition, 4 December, p. 7A.

[nop232-bib-0104] *Times* (1855d) The British Expedition, 17 December p. 7A.

[nop232-bib-0105] *Times* (1855e) The British Expedition, 28 December, p. 7B.

[nop232-bib-0106] *Times* (1856a) Untitled, 14 March, p. 7F.

[nop232-bib-0107] *Times* (1856b) The British Army, 16 May p. 10C.

[nop232-bib-0108] *Times* (1856c) The British Army, 9 June, p. 10B.

[nop232-bib-0109] *Times* (1856d) Mrs Seacole, 5 July, p. 10C.

[nop232-bib-0110] *Times* (1856e) The Dinner to the Guards, 26 August, p. 7C.

[nop232-bib-0111] *Times* (1856f) The Portsmouth Crimean Banquets, 15 September p. 15B.

[nop232-bib-0112] *Times* (1856g) Woolwich Garrison Races, 22 October, p. 10A.

[nop232-bib-0113] *Times* (1856h) The Edinburgh Crimean Banquet, 3 November, p. 10A.

[nop232-bib-0114] *Times* (1856i) Bankruptcy Court. In re Seacole and Day, 7 November, p. 9B.

[nop232-bib-0115] *Times* (1856j) Australia, 24 November, p. 10B.

[nop232-bib-0116] *Times* (1857a) Military and Naval Intelligence, 22 January, p. 7AB.

[nop232-bib-0117] *Times* (1857b) Military and Naval Intelligence, 27 March p. 10C.

[nop232-bib-0118] *Times* (1857c) The Seacole Festival, 30 July, p. 5F.

[nop232-bib-0119] *Times* (1859) The Mails, &c, Southampton, 18 October, p. 6E.

[nop232-bib-0120] *Times* (1863a) Florence Nightingale Fund for the Relief of the Sick and Wounded and the Destitute Families of Polish Patriots, 23 March, p. 8A.

[nop232-bib-0121] *Times* (1863b) Social Science Congress, 13 October, p. 4B.

[nop232-bib-0122] *Times* (1864a) General Garibaldi, 18 April, p. 9C.

[nop232-bib-0123] *Times* (1864b) The Civil War in America, 27 June, p. 9AB.

[nop232-bib-0124] *Times* (1864c) Naval and Military Intelligence, 25 August, p. 9A.

[nop232-bib-0125] *Times* (1864d) National Association for the Promotion of Social Science, 23 September, p. 5A.

[nop232-bib-0126] *Times* (1865) The West India and Pacific Mails, 14 October p. 10G.

[nop232-bib-0127] *Times* (1866) Donations of Stores received, 31 August, p. 6A.

[nop232-bib-0128] *Times* (1867a) The Jamaica Prosecutions, 13 February, p. 12C.

[nop232-bib-0129] *Times* (1867b) United States Sanitary Commission during the War, 20 April, p. 6D.

[nop232-bib-0130] *Times* (1867c) The Wandering Thespians, 18 July, p. 8C.

[nop232-bib-0131] *Times* (1868) The Attempted Assassination of the Duke of Edinburgh, 18 May 1868, p. 5A.

[nop232-bib-0132] *Times* (1869a) The Levant, the Black Sea and the Danube, 3 May, p. 4B.

[nop232-bib-0133] *Times* (1869b) India, 26 October, p. 9C.

[nop232-bib-0134] *Times* (1871) The Distress in and about Paris, 1 February, p. 6B.

[nop232-bib-0135] *Times* (1872a) The Treatment of Prisoners of War, 7 August, p. 3F.

[nop232-bib-0136] *Times* (1872b) The Education of Women, 2 November, p. 6F.

[nop232-bib-0137] *Times* (1873) Life or Death in India, 7 October, p. 6B.

[nop232-bib-0138] *Times* (1876a) Bosnian Fugitives, 13 March, p. 10C.

[nop232-bib-0139] *Times* (1876b) The Atrocities in Bulgaria, 18 September, p. 6.

[nop232-bib-0140] *Times* (1877a) Mr Gladstone at Birmingham, 1 June, p. 10F.

[nop232-bib-0141] *Times* (1877b) The Famine in India, 20 August, p. 6E.

[nop232-bib-0142] *Times* (1881a) Obituary, 21 May, p. 7F.

[nop232-bib-0143] *Times* (1881b) The London Workhouses, 26 December, p. 8B.

[nop232-bib-0144] *United Service Magazine* (1863) A Trip to Vancouver Island, No. 416, p. 383.

[nop232-bib-0145] Vallée G. , ed. (2006) Florence Nightingale on Health in India. Wilfrid Laurier University Press, Waterloo.

[nop232-bib-0146] *Wanagui Evening Herald* (1867) Untitled, 26 July, p. 2.

[nop232-bib-0147] Wellcome Library for the History and Understanding of Medicine. London, Ms 8995/78.

[nop232-bib-0148] Williams B. (2003) The Life and World of Mary Seacole Heinemann, Oxford.

[nop232-bib-0149] Williams B. (2009) Mary Seacole Heinemann, Harlow.

